# An imidazole functionalized pentameric thiophene displays different staining patterns in normal and malignant cells

**DOI:** 10.3389/fchem.2015.00058

**Published:** 2015-10-07

**Authors:** Karin Magnusson, Hanna Appelqvist, Artur Cieślar-Pobuda, Marcus Bäck, Bertil Kågedal, Jon A. Jonasson, Marek J. Los, K. Peter R. Nilsson

**Affiliations:** ^1^Division of Chemistry, Department of Physics, Chemistry and Biology, Linköping UniversityLinköping, Sweden; ^2^Division of Cell Biology, Department of Clinical and Experimental Medicine, Linköping UniversityLinköping, Sweden; ^3^Institute of Automatic Control, Silesian University of TechnologyGliwice, Poland; ^4^Division of Clinical Chemistry, Department of Clinical and Experimental Medicine, Linköping UniversityLinköping, Sweden; ^5^Division of Clinical Pathology and Clinical Genetics, Department of Clinical and Experimental Medicine, Linköping UniversityLinköping, Sweden

**Keywords:** oligothiophenes, fluorescence, cells, imaging, imidazole

## Abstract

Molecular tools for fluorescent imaging of cells and their components are vital for understanding the function and activity of cells. Here, we report an imidazole functionalized pentameric oligothiophene, p-HTIm, that can be utilized for fluorescent imaging of cells. p-HTIm fluorescence in normal cells appeared in a peripheral punctate pattern partially co-localized with lysosomes, whereas a one-sided perinuclear Golgi associated localization of the dye was observed in malignant cells. The uptake of p-HTIm was temperature dependent and the intracellular target was reached within 1 h after staining. The ability of p-HTIm to stain cells was reduced when the imidazole side chain was chemically altered, verifying that specific imidazole side-chain functionalities are necessary for achieving the observed cellular staining. Our findings confirm that properly functionalized oligothiophenes can be utilized as fluorescent tools for vital staining of cells and that the selectivity toward distinct intracellular targets are highly dependent on the side-chain functionalities along the conjugated thiophene backbone.

## Introduction

Fluorescence based cellular imaging is essential for visualizing the localization and the dynamics of cellular compartments and molecular processes (Weijer, 2003; Giepmans et al., 2006; Pittet and Weissleder, 2011; Germain et al., 2012). Therefore, the development of fluorescent tools for imaging cells and their components is crucial. Most fluorescent cell imaging techniques rely on externally applied reporter systems, such as fluorophore labeled antibodies, or endogenously expressed fluorescent, and epitope-tagged fusion partners. For the latter the discovery and development of fluorescent proteins have revolutionized fundamental research within molecular biology (Chalfie et al., 1994). However, these conventional fluorescent tools display inherent limitations, such as low photo-bleaching thresholds or stability, that might restrict their effectiveness for fluorescent imaging of cells (Medintz et al., 2005). Consequently, expanding the toolbox of fluorescent dyes that could be utilized for real-time imaging of cells is of great interest.

Conjugated poly- and oligomers (CPs and COs) have emerged as functional materials for optoelectronic applications, such as light-emitting diodes (Burroughes et al., 1990; Gustafsson et al., 1992; Huang et al., 2010), solar cells (Günes et al., 2007; Kim et al., 2007), transistors (Yan et al., 2009; Hoven et al., 2010), and biosensors (Charych et al., 1993; Chen et al., 1999; Nilsson and Inganäs, 2003; Dore et al., 2004). From a biological perspective, conjugated poly- and oligoelectrolytes (CPEs and COEs) with water compatible side-chain functionalization have also been employed as novel tools for fluorescent imaging of protein aggregates that are associated with numerous neurodegenerative diseases (Sigurdson et al., 2007; Klingstedt et al., 2011, 2013), as well as for distinct cellular elements (Pu et al., 2010; Ding et al., 2011; Pu and Liu, 2011; Feng et al., 2012; Li and Liu, 2012; Cieślar-Pobuda et al., 2014; Gwozdzinska et al., 2014; Magnusson et al., 2015). Due to their electronically delocalized conjugated backbones, CPEs and COEs exhibit specific intrinsic fluorescent characteristics and offer the possibility to use a variety of fluorescent imaging techniques, as well as different modes of detection, such as excitation- and emission-spectra, as well as fluorescent decay (Magnusson et al., 2014). However, CPEs are generated from random polymerization of different monomeric building blocks that renders polydispersed materials with randomized positioning of the side chain functionalities and this lack of chemical precision might limit their performance for cellular imaging. In this regard, COEs having a chemically defined conjugated backbone with an exact number of repetitive units and distinct side-chain functionalities along the conjugate backbone have been generated (Klingstedt et al., 2011; Cieślar-Pobuda et al., 2014; Gwozdzinska et al., 2014). It has been suggested that the chemical nature of the pendant groups along the conjugated backbone determines the cell stainability of pentameric oligothiophenes (Cieślar-Pobuda et al., 2014). From a library of amino acid functionalized pentameric thiophenes, COEs with imidazole moieties were identified as potential staining reagents for cancer cells using flow cytometry (Cieślar-Pobuda et al., 2014). Hence, selectivity toward distinct cellular targets in specific cells might be afforded by proper side-chain functionalization of the conjugated thiophene backbone.

**Figure 1:**
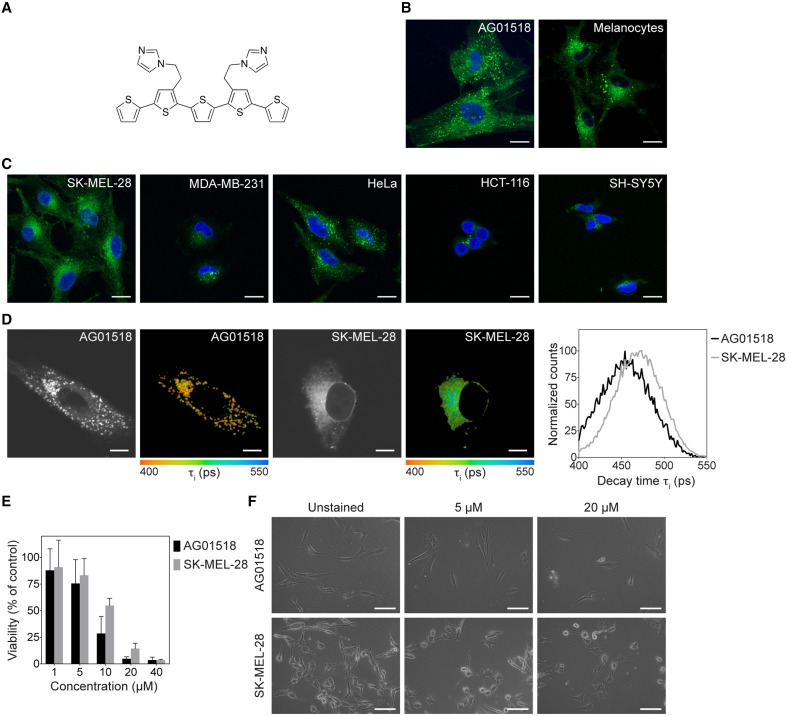
**Chemical structure of p-HTIm and fluorescence images of p-HTIm staining in normal and malignant cells. (A)** The chemical structure of p-HTIm. Fluorescence images of **(B)** normal cells: human fibroblasts (AG01518) and melanocytes, and **(C)** malignant cells: melanoma cells (SK-MEL-28), breast cancer cells (MDA-MB-231), cervical cancer cells (HeLa), colon cancer cells (HCT-116), and neuroblastoma cells (SH-SY5Y) stained with p-HTIm. p-HTIm staining is observed in green and cell nuclei were counterstained with DAPI (blue). Scale bars 20 μm. **(D)** Fluorescence lifetime images and lifetime decay curves of human fibroblasts and melanoma stained with p-HTIm. The contrast-images, as well as the color-coded images according to the fluorescence decay of p-HTIm verified the difference in intracellular localization of the dye. The life time decay curves (right) were also slightly different for the two cell lines. Scale bars represent 10 μm. **(E)** Cell viability as measured by the MTT assays of human fibroblasts and melanoma cells stained with p-HTIm at different concentrations (1–40 μM). **(F)** Phase contrast images of cells 24 h after staining. Scale bars 100 μm. If nothing else stated, cells were stained with 5 μM p-HTIm for 30 min and then incubated in fresh medium for 24 h prior to analysis.

Herein, we applied an imidazole functionalized pentameric thiophene, denoted p-HTIm (Figure 1A) for fluorescent imaging of cells. As it was recently shown that the polydispersed anionic CPE, polythiophene acetic acid (PTAA), could be utilized for differential vital staining of normal fibroblasts and melanoma cells (Magnusson et al., 2015), the primary focus was to assess the utilization of p-HTIm as a novel chemically defined fluorescent tool for visualization of normal and malignant cells. This work confirmed that p-HTIm could be used for staining of distinct cellular compartments in living cells without major influence on cell viability or proliferation. In addition, the staining pattern was dissimilar in normal cells compared to malignant cells. p-HTIm displayed a temperature dependent uptake mechanism in the cells and subtle changes in the chemical composition of the imidazole side chain functionalities were shown to reduce or eliminate the dyes capacity for cell staining. Overall, these studies verified that p-HTIm could be used for staining of living cells and these findings might also aid in the chemical design of optimal ligands for recognizing specific intracellular compartments.

## Materials and methods

### Synthesis of oligothiophenes

**Scheme 1:**
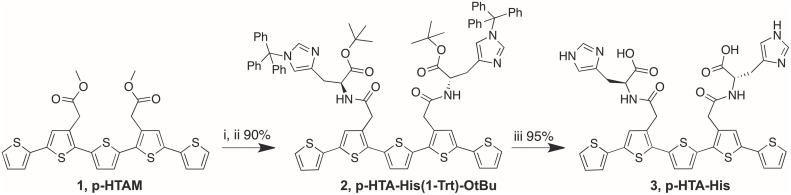
**Reagents and conditions**. (i) 1 M NaOH, dioxane, H_2_O; (ii) H-His(1-Trt)-OtBu, DIPEA, *O*-(7-azabenzotriazol-1-yl)-*N,N,N′,N′*-tetramethyluronium hexafluorophosphate (HATU), DMF; (iii) Et_3_SiH, TFA, DCM.

The synthesis of p-HTIm, p-HTMI, p-HTA-Histamine, p-HTAA, and HS-42 has been described elsewhere (Aslund et al., 2009; Cieślar-Pobuda et al., 2014; Simon et al., 2014). The synthetic route for p-HTA-His is described below and in Scheme 1. All the LCOs were dissolved in deionized water to a concentration of 1.5 mM.

#### Synthesis of p-HTA-His(1-Trt)-OtBu (2)

To a solution of p-HTAM (Aslund et al., 2009) (**1**) (93 mg, 0.167 mmol) in dioxane (2 mL) and H_2_O (0.5 mL) was added 1 M NaOH (0.340 mL, 0.340 mmol). The solution was heated at 65°C for 1 h, neutralized with 1 M HCl, and the solvents were co-evaporated with toluene. The residual was dissolved in DMF (3 mL) and H-His(1-Trt)-OtBu (302 mg, 0.666 mmol) and DIPEA (0.100 mL, 1.04 mmol) were added. The temperature of the solution was lowered to 0°C and *O*-(7-azabenzotriazol-1-yl)-*N,N,N′,N′*-tetramethyluronium hexafluorophosphate (HATU; 190 mg, 0.500 mmol) was added. The solution was allowed to stir for 30 min at 0°C and then for 4 h at room temperature. EtOAc, toluene and brine were added and the organic phase was separated and washed with saturated NH_4_Cl (aq), saturated NaHCO_3_ (aq) and H_2_O, dried, filtered, and concentrated. FC (toluene/EtOAc 1:1+1% TEA gave p-HTA-His(1-Trt)-OtBu (**2**) (90%) as yellowish oil. ^1^H NMR (300 MHz, CDCl_3_) δ 1.32 (s, 18H), 2.86–3.01 (m, 4H), 3.64 (d, *J* = 16.5 Hz, 2H), 3.70 (d, *J* = 16.5 Hz, 2H), 4.60–4.69 (m, 2H), 6.49 (d, *J* = 1.2 Hz, 2H), 6.92 (dd, *J* = 3.6, 5.1 Hz, 2H), 6.98–7.04 (m, 12H), 7.05 (dd, *J* = 1.1, 3.6 Hz, 2H), 7.12 (dd, *J* = 1.1, 5.1 Hz, 2H), 7.14–7.17 (m, 6H), 7.20–7.35 (m, 18H), 7.78–7.84 (b, 2H); ^13^C NMR (75.5 MHz, CDCl_3_) δ 28.2, 29.5, 37.4, 53.3, 75.3, 81.3, 119.3, 123.9, 124.7, 127.6, 127.9, 128.1, 129.9, 132.1, 132.2, 135.5, 136.1, 136.6.

#### Synthesis of p-HTA-His (3)

pHTA-His(1-Trt)-OtBu (**2**) (35 mg, 0.025 mmol) was dissolved in DCM (1 mL) and Et_3_SiH (0.035 mL, 0.414 mmol) was added. TFA (1 mL) was added and the solution was stirred for 3 h. The completeness of the reaction was validated by HPLC-MS. Solvents were co-evaporated with toluene. Purification by HPLC-MS gave p-HTA-His (**3**) (95%) as orange solid. ^1^H NMR (300 MHz, (CD_3_)_2_SO) δ 2.88–3.14 (m, 4H), 3.62 (s, 4H), 4.45–4.60 (m, 2H), 7.07 (s, 2H), 7.11 (dd, *J* = 3.6, 5.1 Hz, 2H), 7.23 (s, 2H), 7.29 (s, 2H), 7.30 (dd, *J* = 1.1, 3.6 Hz, 2H), 7.55 (dd, *J* = 1.1, 5.1 Hz, 2H), 8.13 (s, 2H), 8.52–8.59 (b, 2H). ESI-MS m/z 803.1 [(M+H)^+^ calcd. for C_36_H_31_N_6_O_6_S^+^_5_ 802.9].

### Cells and culture conditions

Human skin fibroblasts (AG01518; passages 12–24; Coriell Institute, Camden, NJ, USA), malignant melanoma cells SK-MEL-28 (HTB-72; ATCC, Manassas, VA, USA) and neuroblastoma cells SH-SY5Y (94030304; Sigma-Aldrich, St. Louis, MO, USA) were cultured in Eagle's minimum essential medium (EMEM) GlutaMAX, supplemented with 50 IU/ml penicillin-G, 50 μg/ml streptomycin, and 10% fetal bovine serum (all from Gibco, Paisley, UK). Melanocytes were kindly provided by Petra Wäster and cultured as described previously (Andersson et al., 2001). Human cervical cancer cells HeLa (CCL-2; ATCC), breast cancer cells MDA-MB-231 (HTB-26; ATCC) and human colon cancer HCT-116 (CCL-247; ATCC) were cultured in Dulbecco's modified eagle medium GlutaMAX supplemented with 50 IU/ml penicillin-G, 50 μg/ml streptomycin and 10% fetal bovine serum. Cells were incubated in humidified air with 5% CO_2_ at 37°C. The day before experiments, cells were trypsinized and seeded to reach 50% confluence. For microscopical examination cells were seeded on glass coverslips No 1.0.

### Vital staining of cells

Cells were stained with LCOs (1–40 μM) in complete medium for 30 min, 37°C. The superfluous probe was removed and the cells were rinsed three times with PBS and incubated in fresh medium for indicated periods of time. For microscopic evaluation, the cells were rinsed three times with PBS, fixed in 4% paraformaldehyde (PFA; 20 min, 4°C), mounted using Vectashield with DAPI (Vector Laboratories, Burlingame, CA, USA) and examined with a Zeiss confocal microscope, LSM 780 (Carl Zeiss AG, Oberkochen, Germany).

### Co-staining with organelle markers

For staining of mitochondria, stained cells were incubated with MitoTracker Orange CMTMRos (75 nM, 30 min, 37°C; Molecular Probes, Eugene, OR, USA). Cells were then fixed in 4% PFA (20 min, 4°C). For immunostaining, PTAA-stained cells were after fixation permeabilized with 0.1% saponin (Sigma-Aldrich) in PBS containing 5% fetal bovine serum (20 min, room temperature) and incubated for 2 h at room temperature with one of the following monoclonal mouse primary antibodies: Golga2/GM130 (1:250, Novus Biologicals, Littleton, CO, USA), lysosome-associated membrane protein 2 (LAMP-2, 1:100; Southern Biotech, Birmingham, AL, USA), p62 (1:100, BD Biosciences, Franklin Lakes, NJ, USA), or polyclonal anti-rabbit primary antibodies; α-tubulin (1:1000, Abcam, Cambridge, UK), calnexin (1:700, Novus Biologicals), early endosomal antigen-1 (EEA-1, 1:400; Sigma-Aldrich), fibronectin (1:400, Sigma-Aldrich), LC3B (1:100, Novus Biologicals), Niemann-Pick type C1 (NPC1, 1:250; Abcam), peroxisomal membrane protein 70 (PMP70, 1:1000; Molecular Probes), PTEN (1:20, Novus Biologicals), anti-Rab11a (1:200, Abcam), and proteasome 20S (1:100, Abcam). This step was followed by incubation with the appropriate secondary antibodies conjugated to Alexa Fluor 594 (1:400, Molecular Probes) for 1 h. All incubations were done in the dark. Next, the cells were mounted in Vectashield with DAPI and examined with a Zeiss confocal microscope, LSM 780 (Carl Zeiss AG, Germany).

### Fluorescence microscopy of stained cells

Stained and fixed cells were analyzed with an inverted Zeiss (Axio Observer.Z1) LSM 780 microscope equipped with a 32 channel QUASAR GaAsp spectral array detector. A plan-Apochromat 63x/1.40 Oil DIC objective lens was used for the imaging. Cells stained with DAPI and one of the probes were analyzed using lasers at 405 and at 458 nm. For cells that in addition were stained with an antibody connected to Alexa-594, a laser at 595 nm were used for excitation. The mitochondrial studies were made using a laser with excitation at 550 nm. Fluorescence lifetime images were acquired using an inverted Zeiss (Axio Observer.Z1) LSM 780 microscope (Carl Zeiss MicroImaging GmbH, Jena, Germany) equipped with a modular FLIM system from Becker & Hickl. In this setup the emitted photons were routed through the Direct coupling (DC) confocal port of the Zeiss LSM 780 scanning unit and detected by a Becker & Hickl HPM-100-40 hybrid detector. Data were recorded by a Becker & Hickl Simple-Tau 152 system (SPC-150 TCSPC FLIM module) with the instrument recording software SPCM version 9.42 in the FIFO image mode, 256 × 256 pixels, using 256 time channels (Becker & Hickl GmbH, Berlin, Germany). For all acquizitions, a T80R20 main beam splitter was used and the pinhole was set to 20.2 μm. Scanning area was set to 235.7 × 235.7 μm, with a scanning resolution of 512 × 512 pixels. Further a Plan-Apochromat 40 × /1.3 Oil DIC objective lens was used and for excitation the 405 nm laser line with a repetition rate of 50 MHz was used. Data was subsequently analyzed in SPCImage version 3.9.4 (Becker & Hickl GmbH, Berlin, Germany) fitting each of the acquired decay curves to a tri-exponential function and color coded images showing the intensity-weighted mean lifetimes were generated with the same software. In total, 20 cells of the respective cell line were analyzed.

### Flow cytometry

For flow cytometry, the cells were detached by trypsizination, washed with PBS and analyzed using a Gallios flow cytometer (Beckman coulter, Gallios™, USA). The fluorescence was measured by using a 488 nm laser and 525/40 BP filter (FL1). Twenty thousand cells were collected for each sample and the data was analyzed using the software Kaluza.

### Viability analysis

The viability of cell cultures was measured using the 3-(4,5-dimethylthiazol-2-yl)-2,5-diphenyltetrazolium bromide (MTT) reduction assay (Sigma-Aldrich). This method is widely used to assess cytotoxicity and cell viability, and it is currently thought that the amount of MTT formazan is proportional to the number of living cells (van Meerloo et al., 2011). Cells were incubated with 0.5 mg/ml MTT for 2 h at 37°C. Then, the MTT solution was removed and the formazan product was dissolved in dimethyl sulfoxide. The absorbance was measured at 550 nm with a VICTOR X Series Multiple Plate Reader (PerkinElmer, Waltham, MA USA).

### Statistical analysis

Experiments were routinely performed at least three times, and the results are presented as the means and standard deviations of independent samples. Differences between two groups were analyzed by the two-tailed Student's *t*-test and, for more than two groups, by one-way ANOVA with the Bonferroni *post-hoc* test. Statistical calculations were performed using the GraphPad Prism 6 software package. Differences were considered significant when *p* ≤ 0.05.

## Results and discussion

### p-HTIm staining of cells

As p-HTIm (Figure 1A) was recently reported to label cells with high intensity (Cieślar-Pobuda et al., 2014), the probe was applied on normal fibroblasts and melanocytes as well as on a panel of five human malignant cell lines (melanoma, breast cancer, cervical cancer, colon cancer, and neuroblastoma). Live cells were stained with 5 μM p-HTIm in cell culture medium for 30 min and then incubated in fresh medium for 24 h prior to analysis. As shown in Figure 1, all cell types were stained by p-HTIm, although to different extent. In normal fibroblasts and melanocytes, p-HTIm fluorescence was mainly concentrated in a punctate pattern throughout the cytosol (Figure 1B). In contrast, in cancer cells the p-HTIm staining was seen in smaller structures and concentrated in a one-sided perinuclear pattern (Figure 1C). The differential staining was most obvious in fibroblasts and melanoma cells, so these cell lines were chosen for further experiments. In addition to the major staining pattern, a less intense network of thin filaments was also observed in these two cell types. The difference in intracellular location of p-HTIm staining between these cell lines was also verified with fluorescence life time imaging microscopy (FLIM). As seen in the contrast images and the color coded images according to the fluorescence decay of p-HTIm, the localization of the probe was different in the two cell types. In addition, a variation in fluorescence life time decays from the dye was also observed in the fibroblasts compared to the melanoma cells (Figure 1D). p-HTIm displayed a shorter lifetime in fibroblasts compared to melanoma cells, indicating different environments of the probe in the two cell types. Hence, p-HTIm could be utilized for differential vital staining of normal cells and malignant cell lines in a similar fashion as the previously reported polydispersed anionic CPE, PTAA (Magnusson et al., 2015).

Next, we investigated the viability of p-HTIm stained cells using the 3-(4,5-dimethylthiazol-2-yl)-2,5-diphenyltetrazolium bromide (MTT) reduction assay. A minor effect on the reducing capacity of stained cell cultures was seen at low concentrations (≤ 5 μM; Figure 1E). However, the morphology of cells revealed no increase in cells undergoing cell death (Figure 1F). Increasing the incubation time up to 72 h did not drastically decrease the viability (Supplementary Material, Figure S1). This, in combination with the fact that cells stained with p-HTIm were viable and retained the same proliferation rate up to 10 generation (data not shown), lead us to conclude that these minor effects on cell viability are negligible. In contrast, increased concentrations of p-HTIm (≥10 μM) induced cell death as detected by a marked loss of reducing capacity and apoptotic morphology of the cells (Figures 1E–F). In addition, at these higher concentrations, the fibroblasts seemed to be more sensitive to the toxicity of p-HTIm than the melanoma cells. From these experiments, we concluded that concentrations ≥10 μM were toxic to both cells lines. Therefore, a staining concentration of 5 μM p-HTIm was used for all further experiments.

### Kinetics of p-HTIm staining

**Figure 2:**
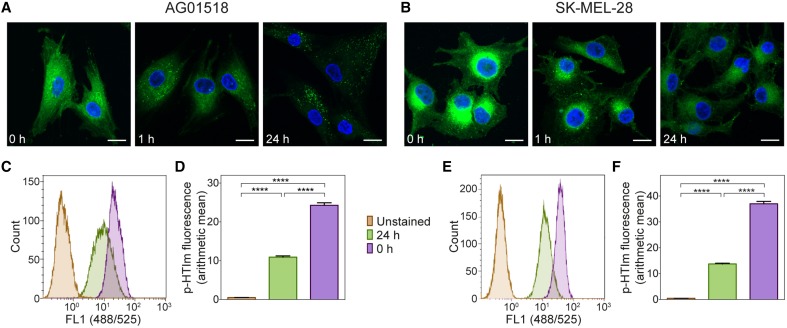
**Kinetic studies of p-HTIm staining**. Fluorescence images of **(A)** human fibroblasts (AG01518) and **(B)** melanoma cells (SK-MEL-28) stained with p-HTIm (5 μM, 30 min; green) and incubated in fresh medium for indicated periods of time. Cell nuclei were labeled with DAPI (blue). Scale bars 20 μm. Histograms of fluorescence intensity from flow cytometry analysis of **(C)** human fibroblasts and **(E)** melanoma cells stained with p-HTIm as described above. Graphs of arithmetic mean fluorescence intensity of **(D)** human fibroblasts (calculated from **C**) and **(F)** melanoma cells (calculated from **E**), *n* = 4. Significant differences were determined by one-way ANOVA with Bonferroni *post-hoc* test, ^****^p ≤ 0.0001.

Next, the kinetics of the p-HTIm staining in fibroblasts and melanoma cells was analyzed. By evaluating the staining pattern at different periods of time after the staining procedure (5 μM, 30 min), the intracellular location of p-HTIm could be assessed. In fibroblasts, p-HTIm was more or less evenly distributed in the cytoplasm directly after staining and after 1 h a punctate pattern similar to the arrangement observed after 24 h appeared (Figure 2A). In melanoma cells, the one-sided perinuclear pattern was already seen directly after staining and the pattern persisted after 24 h of incubation (Figure 2B). However, the intensity of the p-HTIm fluorescence was decreasing over time in both cell lines and this change was verified by flow cytometry (Figures 2C–F).

**Figure 3:**
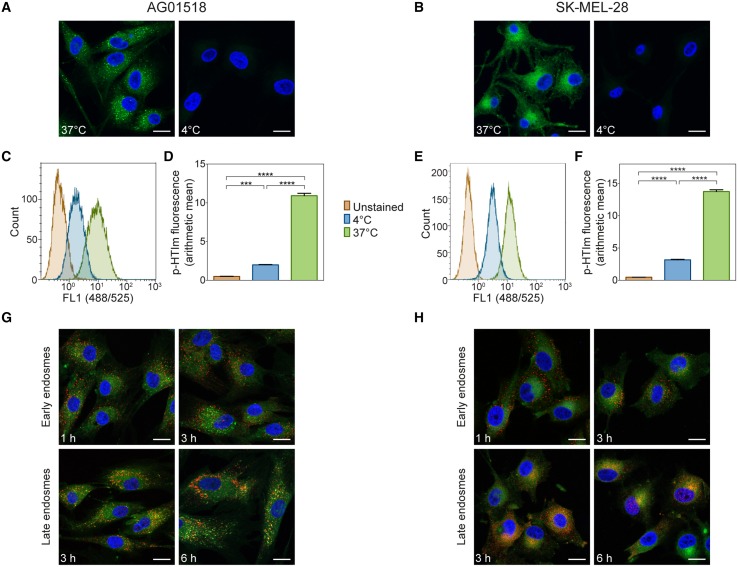
**Analysis of p-HTIm uptake**. Fluorescence images of **(A)** human fibroblasts (AG01518) and **(B)** melanoma cells (SK-MEL-28) stained with p-HTIm (5 μM, 30 min; green) at 37 or at 4°C and incubated in fresh medium for 24 h. Cell nuclei were counterstained with DAPI (blue). Histograms of fluorescence intensity from flow cytometry analysis of **(C)** human fibroblasts and **(E)** melanoma cells stained with p-HTIm as described above. Graphs of arithmetic mean fluorescence intensity of **(D)** human fibroblasts (calculated from **C**) and **(F)** melanoma cells (calculated from **E**), *n* = 4. Significant differences were determined by one-way ANOVA with Bonferroni *post-hoc* test, ^***^p ≤ 0.001, ^****^p ≤ 0.0001. **(G)** Human fibroblasts and **(H)** melanoma cells were stained with p-HTIm and incubated in fresh medium for indicated periods of times. Colocalization with the endocytic pathway was analyzed by costaining of early endosomes (EEA1) and late endosomes (NPC1). Scale bars 20 μm.

As the kinetic experiments suggested that the cellular uptake of p-HTIm occurs rather fast, we next addressed the mechanism of uptake in more detail. Firstly, the staining procedure was performed at 4°C, where cellular activity and ATP-dependent processes ceases. As seen in Figures 3A–B, incubation at low temperature prevented staining to a large degree in both cell types. These observations were also confirmed by flow cytometry as the intensity of p-HTIm fluorescence was drastically reduced when performing the staining procedure at 4°C (Figures 3C–F). Hence, the uptake of p-HTIm was dependent on cellular activity and most likely ATP-dependent. Secondly, co-staining of proteins of the endocytic pathway and p-HTIm was performed. However, these experiments revealed no co-localization of p-HTIm. Thus, endosomes and consequently, endocytosis was excluded as the major route by which p-HTIm enter cells (Figures 3G,H).

Overall, the kinetic experiments, as well as the uptake studies, suggested that p-HTIm enters both the cell types via a temperature-dependent pathway and that the distinct intracellular targets are reached within 1 h. Interestingly, recent studies of anionic CPEs, have suggested that these polydisperse dyes enter fibroblasts through a temperature independent fibronectin mediated pathway (McRae et al., 2008; Magnusson et al., 2015). Thus, although the punctate staining observed previously for PTAA (Magnusson et al., 2015), it seems that these two thiophene-based dyes, p-HTIm and PTAA, may have, at least partly, different cellular uptake mechanism.

### Intracellular target of p-HTIm

**Figure 4:**
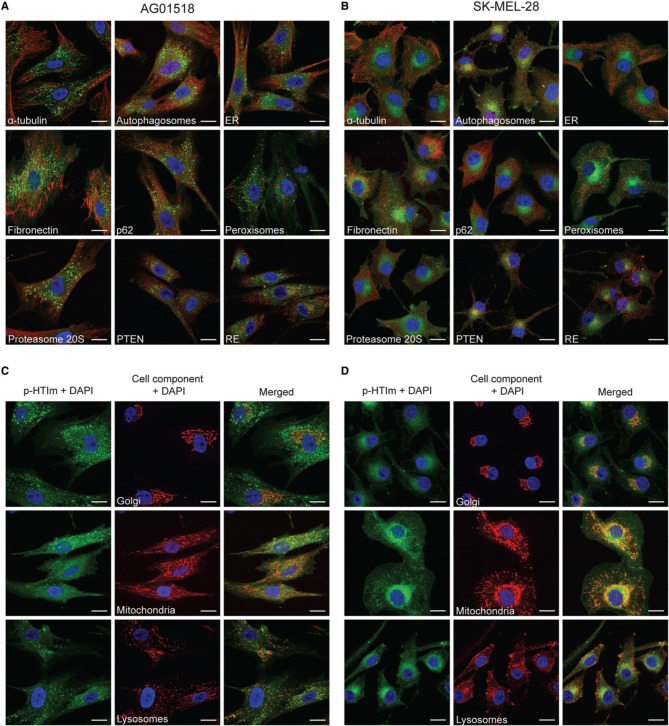
**Intracellular targets of p-HTIm**. Merged fluorescence images of **(A,C)** human fibroblasts (AG01518) and **(B,D)** melanoma cells (SK-MEL-28) stained with p-HTIm (5 μM, 30 min; green) and incubated in fresh medium for 24 h. The cells were stained with fluorescent markers against α-tubulin, autophagosomes (LC3), ER (calnexin), fibronectin, p62, peroxisomes (PMP70), proteasome 20S, PTEN, recycling endosomes (rab11a), Golgi (Golga2), mitochondria (mitotracker), and lysosomes (LAMP-2) (red). Cell nuclei were labeled with DAPI (blue). The color channels are split in (**C**) and (**D**) to improve the visualization. Scale bars 20 μm.

In order to elucidate the intracellular targets of p-HTIm, co-staining with conventional fluorescent markers for intracellular proteins or organelles were performed (Figure 4). No co-localization of p-HTIm and various cellular components such as α-tubulin, autophagosomes, ER, fibronectin, p62, peroxisomes, proteasomes, PTEN, recycling endosomes (RE), or nucleus was detected (Figures 4A,B). Interestingly, in SK-MEL-28 the one-sided perinuclear p-HTIm staining coincides although not co-localizes with the Golgi apparatus (Figure 4D). This observation was confirmed in the other malignant cell lines (Supplementary Material, Figure S2), but was not seen in fibroblasts where the fluorescent punctate pattern was precluded from the Golgi area (Figure 4C). Presumably, the differential staining reflects the fundamental biological difference between normal cells and malignant cells. For the fibroblasts, the punctate p-HTIm pattern showed a partial co-localization with the lysosomal membrane marker LAMP-2 (Figure 4C). In addition, co-staining with mitochondria revealed some co-localization between the less intense p-HTIm network and mitochondria for both cell lines (Figures 4C,D).

Overall, the co-localization experiments verified that the intracellular targets stained by p-HTIm in fibroblasts and melanoma cells are different. Partial co-localization with LAMP-2 in fibroblasts was also observed for the polydispersed anionic CPE, PTAA (Magnusson et al., 2015), suggesting that p-HTIm and PTAA stain a similar intracellular compartment in fibroblasts. Similar to p-HTIm, the intracellular target of PTAA in melanoma cells was also distributed perinuclearly close to one side of the nucleus (Magnusson et al., 2015). However, in contrast to p-HTIm, the PTAA staining was sensitive to detergents, which made co-staining with antibodies impossible (Magnusson et al., 2015), suggesting that p-HTIm might have a higher affinity for the Golgi associated intracellular target than PTAA. Thus, having imidazole, p-HTIm, instead of acetic acid, PTAA, side-chain functionalities along the conjugated thiophene backbone might be vital for achieving the superior intracellular staining observed for p-HTIm.

### Staining of cells with p-HTIm and PTAA analogs

**Figure 5:**
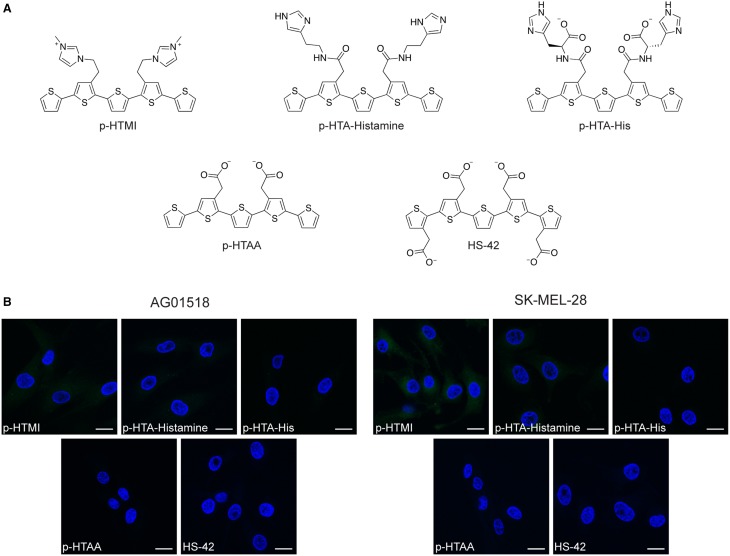
**Chemical structures and fluorescence images of cells stained with analogs to p-HTIm and polythiophene acetic acid (PTAA). (A)** Chemical structures of analogs to p-HTIm: p-HTMI, p-HTA-Histamine and p-HTA-His and analogs to PTAA: p-HTAA and HS-42. **(B)** Fluorescence images of human fibroblasts (AG01518) and melanoma cells (SK-MEL-28) stained with one of the dyes above (5 μM, 30 min; green) and incubated in fresh medium for 24 h. Cell nuclei were labeled with DAPI (blue). Scale bars 20 μm.

To further investigate the correlation between the imidazole side chain functionalities and the observed cellular staining of p-HTIm, three chemically related analogs to p-HTIm were tested toward the fibroblasts and melanoma cells (Figure 5A). p-HTMI has a methylated imidazole functionality that renders a positively charged side chain, whereas p-HTA-Histamine and p-HTA-His have side chain functionalities resembling histamine or histidine, which both contain an imidazole functionality. In addition, p-HTAA and HS-42, two pentameric oligothiophenes having acetic acid side-chain functionalities resembling the anionic polydispersed PTAA (Magnusson et al., 2015) were included (Figure 5A). As shown in Figure 5B, with the same staining procedure (5 μM dye for 30 min and incubation in fresh medium for 24 h) as for p-HTIm, neither of these analogs revealed any significant staining of the fibroblasts or melanoma cells. From a molecular perspective, p-HTA-Histamine and p-HTA-His also display imidazole motifs, but these are slightly different compared to the nitrogen heterocyclic side-chain functionalities of p-HTIm. Firstly, the spacing between the imidazole motifs and the conjugated thiophene backbone are different, as these dyes have an additional peptide bond separating the thiophene ring and the imidazole moiety. Secondly, in contrast to p-HTIm, tautomeric forms of the imidazole functionality can exist for p-HTA-Histamine and p-HTA-His, as the proton can be localized on either of the two nitrogen atoms. This tautomerization of the imidazole moiety is prevented for p-HTIm, since one of nitrogen atoms is utilized for attaching the imidazole ring to the thiophene ring via an ethylene linker. Consequently, for p-HTIm only one of the nitrogens in the imidazole moiety can be protonated. Interestingly, methylation of this nitrogen, rendering p-HTMI, also decreased the staining. However, when using higher concentrations of p-HTMI (20 μM), some staining was observed (Supplementary Material, Figure S3). Overall, these experiments verified that minor chemical modifications of the imidazole side chain functionalities diminished or abolished the staining observed with p-HTIm. Thus, the intracellular staining pattern observed for p-HTIm was highly dependent on the imidazole side chain functionalities.

Similar to the other pentameric oligothiophene analogs, p-HTAA, and HS-42 did not show any considerable staining of the cells (Figure 5B). Negatively charged molecules are typically not readily transported into living cells and a delay in cellular uptake in fibroblasts has been observed for anionic CPEs (McRae et al., 2008; Magnusson et al., 2015). Thus, due to their carboxyl acid functionalities p-HTAA and HS-42 might not enter the cells. However, similar oligothiophenes have been utilized for vital staining of intra-cellular protein aggregates in cell models and transgenic mice (Mahajan et al., 2011; Wegenast-Braun et al., 2012; Brelstaff et al., 2015), signifying that anionic oligothiophene can be transported into living cells and identify a selective target. Hence, the absence of p-HTAA and HS-42 staining of fibroblasts and melanoma cells might also be due to the lack of a proper intra-cellular target for these anionic oligothiophenes. In addition, the polymeric version of thiophene acetic acid, PTAA, clearly stained intracellular compartments in fibroblasts and melanoma cells (Björk et al., 2007; Magnusson et al., 2015), suggesting that the length of the conjugated thiophene backbone is also a chemical determinant that will influence the probe's ability to stain cells. A recent study of phenylenevinylene conjugated oligoelectrolytes also showed that the dimensions of the conjugated phenylene vinylene core appeared to play a role in the toxicity and cell staining profile of these ligands (Gwozdzinska et al., 2014). All together our results demonstrate a clear structure activity relationship between the side chain functionalization along the pentameric thiophene backbone and the capacity of the fluorescent ligand for staining intracellular targets.

Apparently, pentameric thiophenes can be properly functionalized with distinct side chain functionalities and be utilized as fluorescent tools for real-time imaging of distinct cellular compartments in specific cells. In comparison to conventional fluorescent tools, the thiophene based dyes display rather low photo-bleaching and good stability (Cieślar-Pobuda et al., 2014). Furthermore, in contrast to antibodies, these small molecular dyes preferably interact with a specific molecular motif, such as protein aggregates with repetitive β-sheet structure (Sigurdson et al., 2007; Klingstedt et al., 2011, 2013), rather than a distinct protein. Thus, novel biological phenomenon underlying the molecular differences between normal and malignant cells might be revealed. However, to realize the full potential of pentameric thiophenes for studying these fundamental biological differences, it would be essential to determine the specific molecular entity targeted by a particular pentameric thiophene. In this regard, an azide-functionalized pentameric thiophene that could be utilized for cupper free click chemistry was recently presented (Johansson et al., 2015). If a similar azide functionality can be introduced on p-HTIm, the intra-cellular molecular target detected by p-HTIm can be isolated and characterized by biochemical techniques. Such experiments are ongoing in our laboratory and resolving the molecular target for an individual pentameric thiophene with specific side-chain functionalities will also be vital for further chemical improvement of novel thiophene based dyes for cell imaging.

## Conclusions

In conclusion, the imidazole functionalized pentameric oligothiophene, p-HTIm, was identified as a fluorescent ligand displaying different staining patterns in normal and malignant cells. The superior functionality of p-HTIm compared to structurally related compounds could be assigned to the imidazole side-chain functionalities along the conjugated backbone. We foresee that our findings will aid in the chemical design of oligothiophene ligands that could be utilized for exploring differences between normal and malignant cells.

## Conflict of interest statement

The authors declare that the research was conducted in the absence of any commercial or financial relationships that could be construed as a potential conflict of interest.
