# Supplementary material for: An imidazole functionalized pentameric thiophene displays different staining patterns in normal and malignant cells
Source: Front Chem. 2015 Oct 7;3:58. doi: 10.3389/fchem.2015.00058 (PMC4595803; doi:10.3389/fchem.2015.00058)

Supplementary Material

An imidazole functionalized pentameric thiophene displays different staining patterns in normal and malignant cells

Karin Magnusson, Hanna Appelqvist, Artur Cieślar-Pobuda, Marcus Bäck, Bertil Kågedal, Jon Jonasson, Marek J. Los, and K. Peter R. Nilsson*

*** Correspondence:** K. Peter R. Nilsson: E-mail: petni@ifm.liu.se

1 Supplementary Figures and NMR spectra

1.1 Supplementary Figures

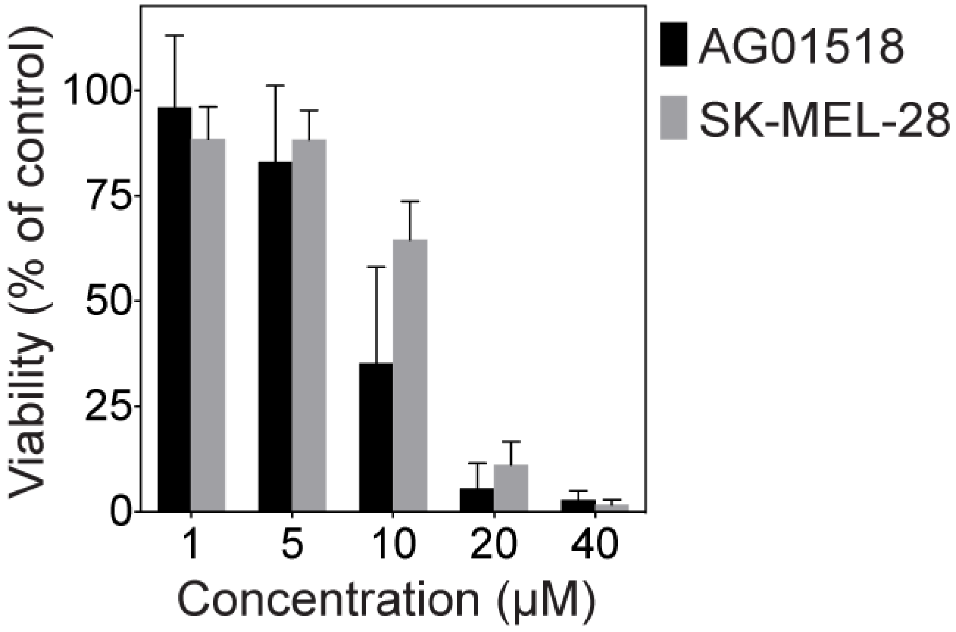

Figure S1. Cell viability as measured by the MTT assays of human fibroblasts (AG01518) and melanoma cells (SK-MEL-28) stained with p-HTIm at different concentrations. The cells were stained with the respective concentration of p-HTIm for 30 min and then incubated in fresh medium for 72 h prior to analysis.

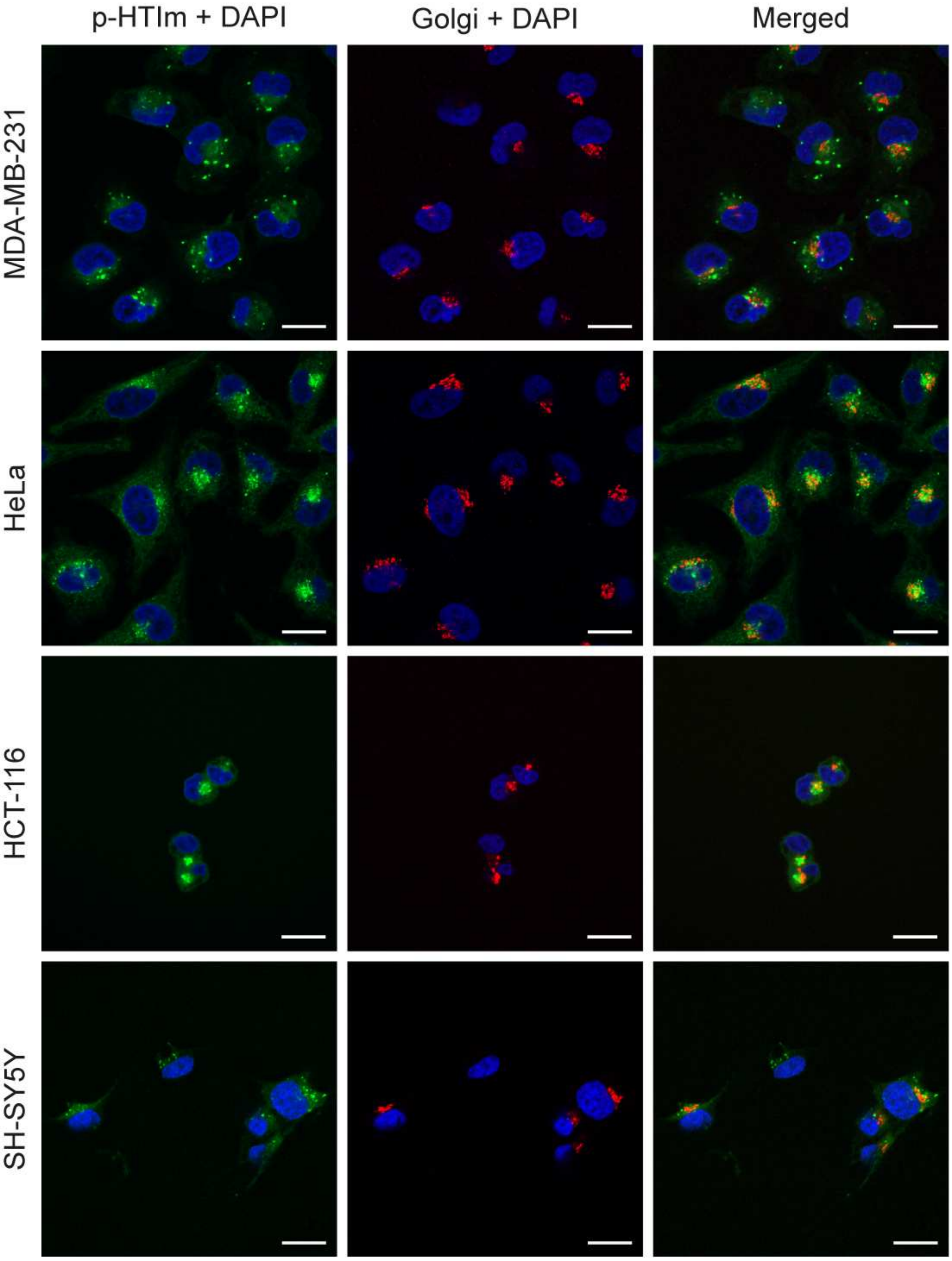

Figure S2. Co-staining with p-HTIm and Golgi. Fluorescence images of breast cancer cells (MDA-MB-231), cervical cancer cells (HeLa), colon cancer cells (HCT-116) and neuroblastoma cells (SH-SY5Y) stained with p-HTIm (5 µM, 30 min; green) and incubated in fresh medium for 24 h. After fixation the cells were stained with a fluorescent marker against Golgi (Golga2; red) and cell nuclei were labelled with DAPI (blue). Scale bars 20 µm.

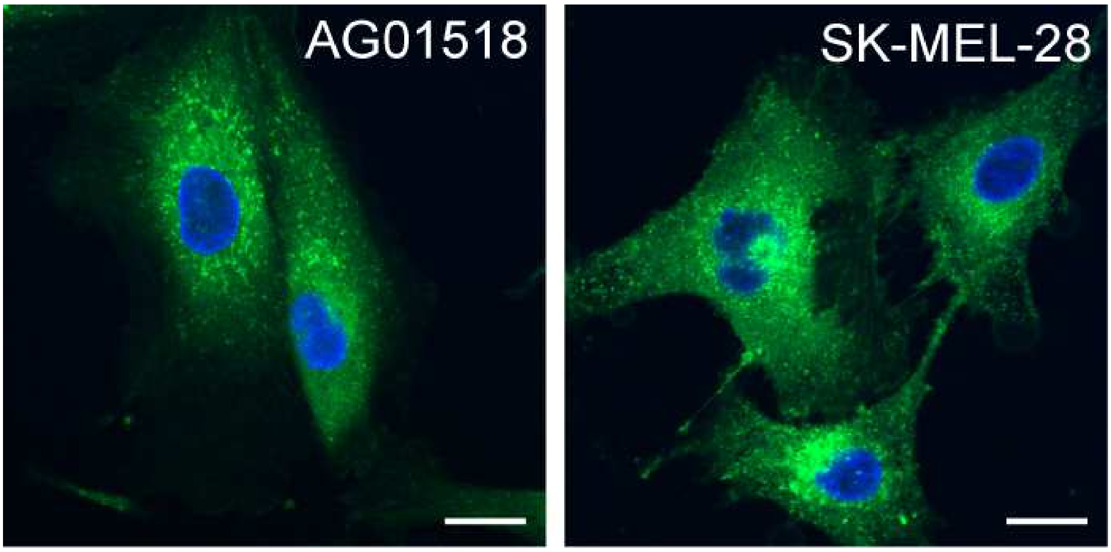

Figure S3. Fluorescence images of cells stained with p-HTMI. Fluorescence images of human fibroblasts (AG01518) and melanoma cells (SK-MEL-28) stained with p-HTMI (20 µM, 30 min; green) and incubated in fresh medium for 24 h. Cell nuclei were labeled with DAPI (blue). Scale bars 20 µm.

1.2 NMR spectra

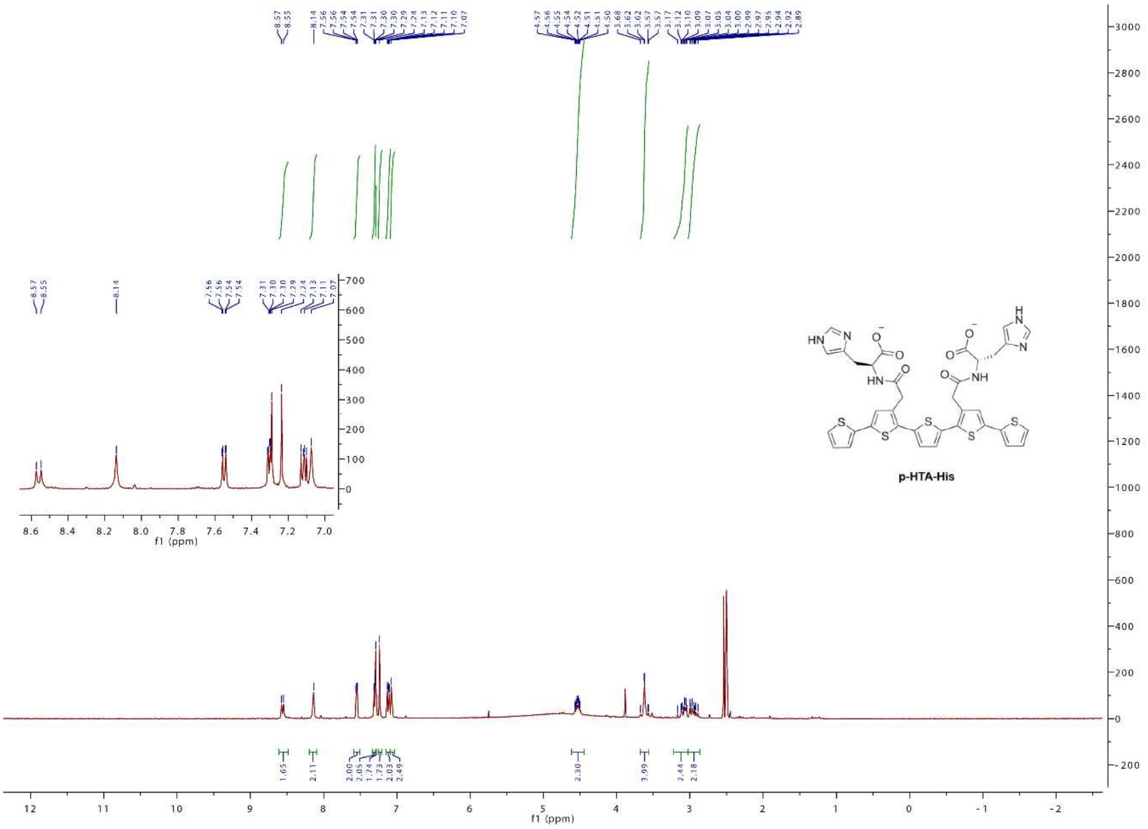

Supplement: Supplementary file 1 [file Presentation1.PDF]
